# Monitoring extreme meteo-marine events in the Mediterranean area using the microseism (Medicane Apollo case study)

**DOI:** 10.1038/s41598-022-25395-9

**Published:** 2022-12-09

**Authors:** Alfio Marco Borzì, Vittorio Minio, Flavio Cannavò, Angelo Cavallaro, Sebastiano D’Amico, Adam Gauci, Raphael De Plaen, Thomas Lecocq, Gabriele Nardone, Arianna Orasi, Marco Picone, Andrea Cannata

**Affiliations:** 1 Dipartimento di Scienze Biologiche, Geologiche ed Ambientali - Sezione di Scienze della Terra, Università degli Studi di Catania, Catania, Italy; 2 Istituto Nazionale di Geofisica e Vulcanologia - Sezione di Catania, Osservatorio Etneo, Catania, Italy; 3 Ac2 S.R.L., Zafferana Etnea, Italy; 4 Department of Geosciences, University of Malta, Msida, Malta; 5 Seismology-Gravimetry, Royal Observatory of Belgium, Brussel, Belgium; 6 Centro Nazionale per la Caratterizzazione Ambientale e la Protezione della Fascia Costiera, la Climatologia Marina e l’Oceanografia Operativa, Italian National Institute for Environmental Protection and Research, Rome, Italy

**Keywords:** Atmospheric science, Climate change, Ocean sciences, Atmospheric dynamics, Seismology, Geophysics

## Abstract

Microseism is the continuous background seismic signal caused by the interaction between the atmosphere, the hydrosphere and the solid Earth. Several studies have dealt with the relationship between microseisms and the tropical cyclones, but none focused on the small-scale tropical cyclones that occur in the Mediterranean Sea, called Medicanes. In this work, we analysed the Medicane Apollo which impacted the eastern part of Sicily during the period 25 October–5 November 2021 causing heavy rainfall, strong wind gusts and violent sea waves. We investigated the microseism accompanying this extreme Mediterranean weather event, and its relationship with the sea state retrieved from hindcast maps and wave buoys. The spectral and amplitude analyses showed the space–time variation of the microseism amplitude. In addition, we tracked the position of Apollo during the time using two different methods: (i) a grid search method; (ii) an array analysis. We obtained a good match between the real position of Apollo and the location constraint by both methods. This work shows that it is possible to extract information on Medicanes from microseisms for both research and monitoring purposes.

## Introduction

During the period 25 October–5 November 2021, the eastern part of Sicily, and especially the areas between Catania, Messina and Siracusa, was impacted by a low-pressure system, that on 28 October acquired the characteristics of a Medicane (MEDIterranean hurriCANE) or tropical-like cyclone (TLC), called Apollo.

Medicanes genesis is favoured when an extratropical depression gets isolated from the polar jet stream. This “cut-off” feature becomes quasi-stationary above the Mediterranean Sea and can use the heat and humidity largely available from the sea to produce organised convection^1^. Medicanes have features similar to the tropical cyclones, both when observed on satellite images and considering their dynamic and thermodynamic characteristics. They are characterized by the presence of an “eye”, a warm-core anomaly that is maximum near the surface, a strong rotation around the pressure minimum, an eyewall with convective cells, from which rain bands extend, inducing sea-level rise, storm surge and sea waves that can reach significant heights of about five meters^2^. Unlike tropical cyclones, however, the lifetime of the Medicanes is restricted to a few days, due to the limited extent of the Mediterranean Sea, their main source of energy. They also only reach fully tropical characteristics for a short period of time, while extratropical features prevail for most of their lifetime^3, 4^. The horizontal extent is generally confined to a few hundred km and the intensity rarely exceeds category 1 of the Saffir-Simpson hurricane wind scale^2^. On average we observe only 1–2 events per year and usually, these cyclones are formed from September to January. During this time interval the Mediterranean Sea reaches the maximum temperature (September), and first cold upper-air troughs are observed. Another important parameter is the sea-air temperature gradient^5, 6^. In particular, intense convective instability is triggered when polar jet stream brings cold air masses over the relatively warmer Mediterranean Sea^5, 6^.

Before Apollo, other Medicanes affected the Ionian Sea such as: Numa during November 2017^7^, Zorbas in late September 2018^8–10^ and Ianos in mid-September 2020^11, 12^.

**Figure 1:**
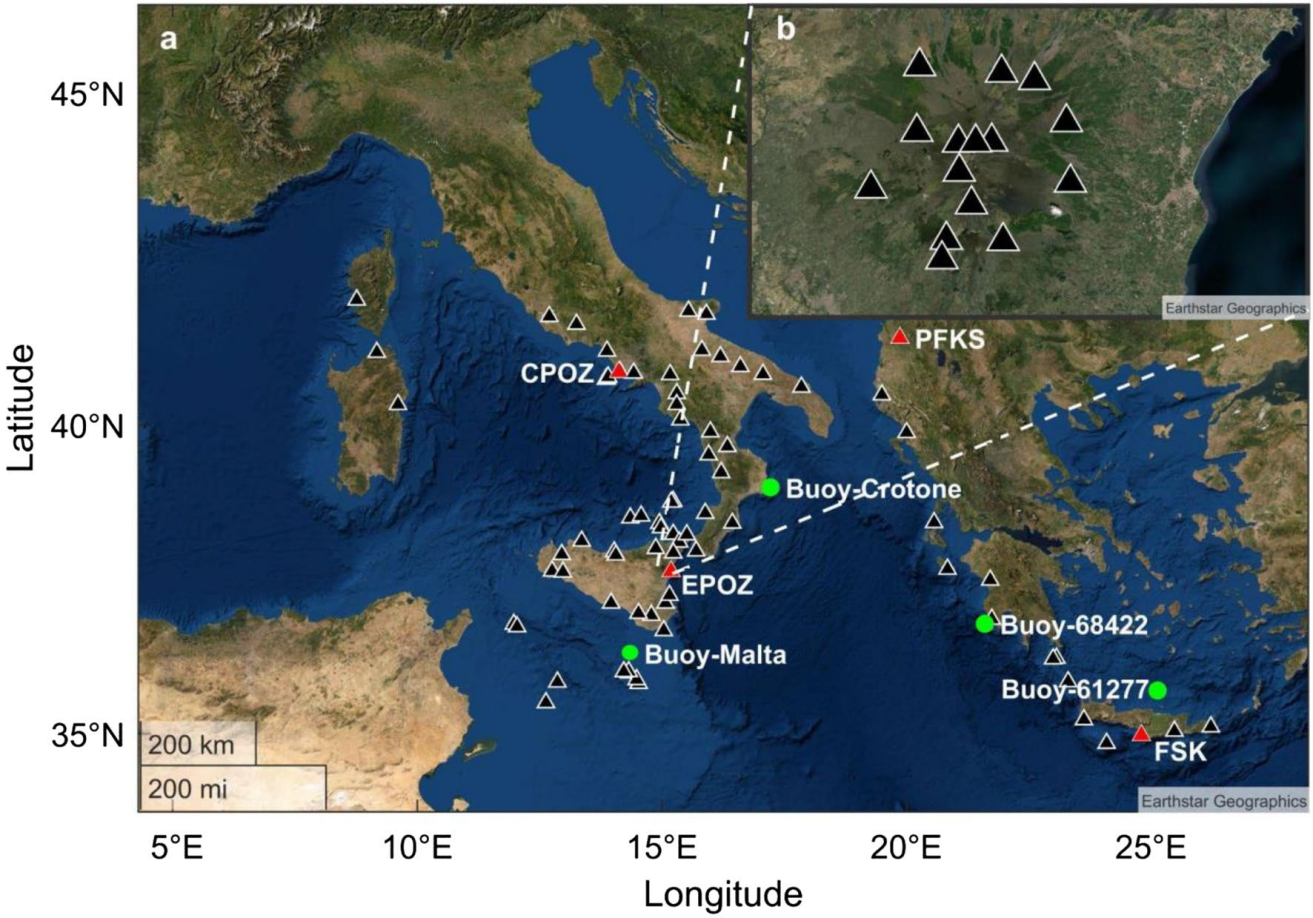
Satellite image of the Mediterranean area with a selection of the broadband seismic stations available in the ORFEUS and INGV databases and used in the spectral analysis and in the grid search method (**a**) and selection of the broadband seismic stations available in the Etna area maintained by INGV-OE (**b**), used in the array analysis. The four wavemeter buoys used in this study are shown with the green dots (base image source ©Earthstar Geographic).

The Medicane Apollo developed on 25 October 2021 near the Libyan coast as a depression vortex. During its northward motion, the vortex became more intense thanks to the high temperature of the Mediterranean Sea, and on 28 October took the features of a Medicane. The effects of the Medicane Apollo were observed particularly near Catania, with a pluviometric-mean of about 200 mm/48 h and with a peak of 448 mm/48 h recorded by the Sicilian Meteorological service (“Regione Siciliana—SIAS—Servizio Informativo Agrometeorologico Siciliano”) near Linguaglossa, and in the area of Siracusa, where the SIAS measured > 200 mm rain on 29 October. The highest wind gusts were measured on the same date (104 km/h) and the minimum pressure was estimated to be 999 hPa^1^. The sea wave activity also showed an intensification with significant wave heights exceeding 3 m as recorded by the ISPRA buoy located at Crotone (Fig. 1a).

After the Medicane transition, the Sicilian regional government declared a state of emergency for 32 municipalities (in the provinces of Catania, Messina, Siracusa and Ragusa) mostly affected by Apollo. The damage caused by Apollo is quantified about 2 millions of euro for what concerns the emergency interventions and about 50 million for agriculture, productive activity and infrastructure. As for the latter, damages were observed along the Catania-Siracusa highway, as a result of the overflow of the Simeto river, and in the ports, as a consequence of the violent wave motions^13^.

The impact of the Medicane on the sea state and in particular the development of violent wave motions causes an energy transfer from the sea waves to the solid Earth. This energy transfer generates the so-called microseism, the most continuous and ubiquitous seismic signal on the Earth, caused by the interaction between the atmosphere, the hydrosphere, and the solid Earth^14, 15^. On the basis of both source mechanism and spectral content, it is possible to divide this signal into primary microseism (PM), secondary microseism (SM), and short period secondary microseism (SPSM)^16^. The PM shows the same spectral content as the oceanic waves with a period between 13 and 20 s, that is associated with the energy transfer of oceanic waves breaking against the shoreline and exhibits low amplitudes^15, 17^. The SM is generated by sea waves with the same frequency traveling in opposite directions, and shows frequency about twice the frequency of the oceanic waves (corresponding with a period of 5–10 s) and amplitude higher than the PM^14, 17–19^. The SPSM is under 5 s period and is generated by the interaction between local wave motions near the coastline^20^.

Several studies showed a correlation between microseism and the sea state^21–24^, and more specifically between microseism and cyclonic activity^20, 25–31^. Bromirski^25^ and Bromirski et al.^20^ highlighted the link between the SM, SPSM and cyclonic activity. Other authors considered the relationship between SM, SPSM and hurricanes^26^, typhoons^28^ and tropical cyclones^31^. In particular, Gerstoft et al.^26^ tracked the position of hurricane Katrina by using the microseism recorded by a large-scale array. More recently, Retailleau and Gualtieri^29^ were able to track the path of the typhoon Ioke in 2006 by using microseism, and Gualtieri et al.^27^ showed how its spectral amplitude has a strong relationship with tropical cyclone intensity.

In addition, it is also possible to analyze extreme meteorological past events for which there are no directly available meteorological data. In fact, Lecocq et al.^32^ digitised old seismograms already proved their potential in providing useful information about the microseismic signal associated with oceanic storms.

In spite of the extensive research on microseism and its relationship with cyclonic activity, the relationships between SM, SPSM, and Medicanes have never been specifically investigated, nor the microseism signature of such Mediterranean extreme meteorological events has ever been explored. For this reason, we approach the study of the microseism recorded during the Medicane Apollo (25 October–5 November 2021) using two different methods, array analysis and amplitude-based grid search, to track the seismic position of Apollo during its lifetime.

## Results and discussion

The period taken into account in this study is 20 October–5 November 2021. This period was chosen to include the development of the Medicane, its climax in terms of wind velocity, precipitation intensity and sea wave height, which took place on 28–29 October 2021, and the subsequent loss of intensity.

We consider the spectrograms, the root mean square (RMS) amplitude time series and the space–time distributions of the microseism amplitude derived from our analysis.

**Figure 2:**
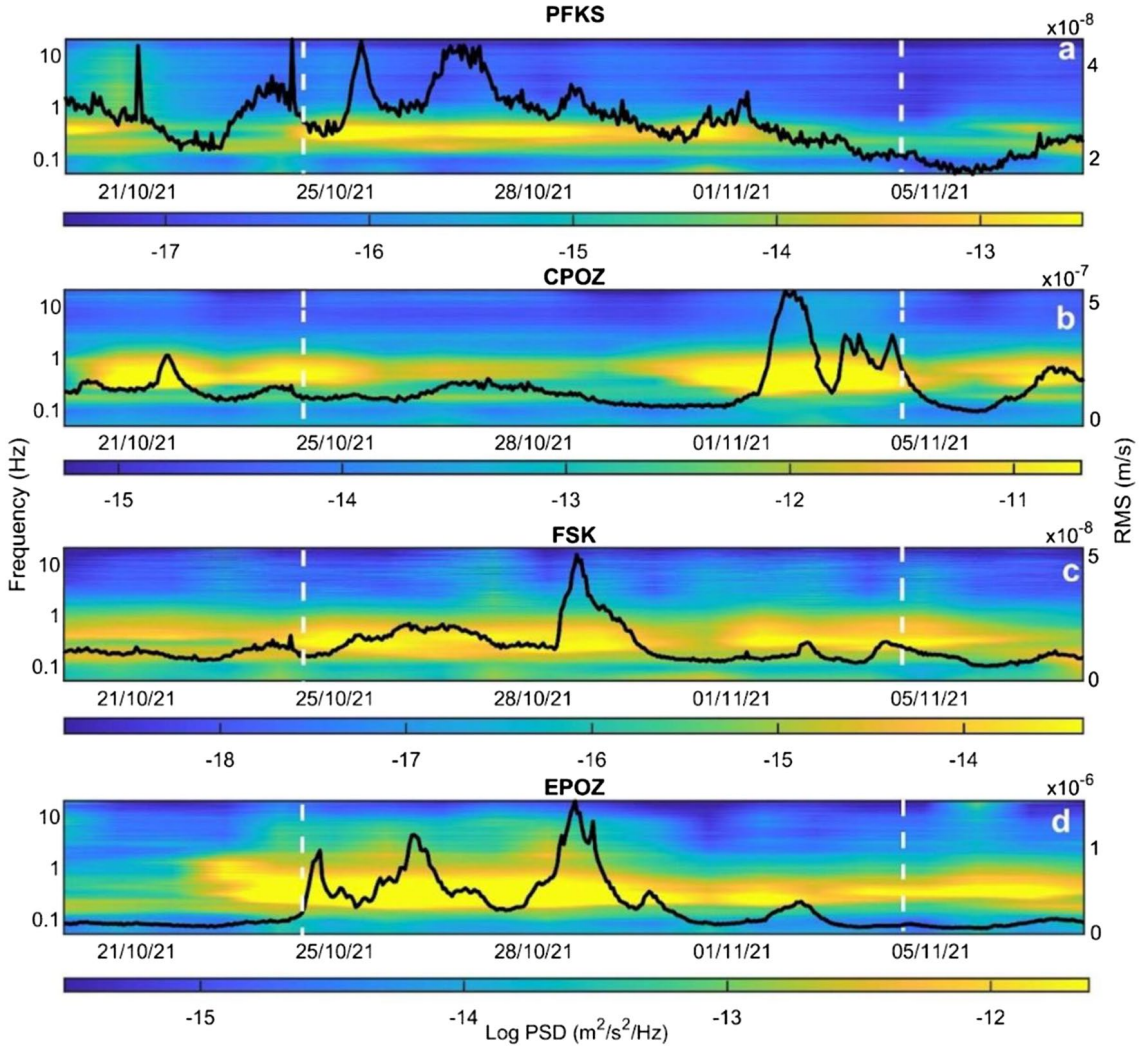
Spectrograms and RMS amplitude time series, for the SM band (0.1-0.2 Hz), of the seismic signal recorded by the vertical component of 4 stations located along the Greek coastline (**a**, **c**), in central Italy (**b**) and in the eastern part of Sicily (**d**) (see Fig. 1a for the station locations).

Spectrograms and RMS amplitude time series were obtained by analysing the vertical components of the seismic signals recorded by 4 stations installed along Greek coastal areas (Fig. 2a,c), in Central Italy (Fig. 2b) and in the eastern part of Sicily (Fig. 2d). These spectrograms highlight that a large portion of the energy is focused in the band 0.1–1 Hz, that partly corresponds to the microseism. Furthermore, it is worth noting that EPOZ, PFKS and FSK (three stations installed close to the Ionian Sea area) show the maximum energy during the period of the Medicane Apollo (highlighted by the dashed vertical lines in Fig. 2), while CPOZ, installed close to the Tyrrhenian coastline, shows a different behaviour with maximum amplitude during distinct time intervals.

In the RMS amplitude time series, we observe different characteristics for each station and microseism band taken into account in our work. The highest values of RMS amplitude can be found in SM (Fig. 2) and SPSM (Supplementary Fig. 1) bands, and the lowest values in the PM (Supplementary Fig. 2) band, showing that the correlation between microseism and cyclonic activity is clear in the SM and SPSM bands while is absent in the PM band, as already discussed in the literature^20, 25–31^. Similarly to what we saw in the spectrograms, if we consider each station individually, we can observe that EPOZ, the station nearest to the cyclone eye, shows the highest values of RMS amplitude (Fig. 2d), and the stations PFKS and FSK, more distant than EPOZ but installed in the Ionian area, show lower values of RMS amplitude with a similar pattern (Fig. 2a,c, respectively), while CPOZ exhibits a different trend with minimum values during the Medicane (Fig. 2b).

**Figure 3:**
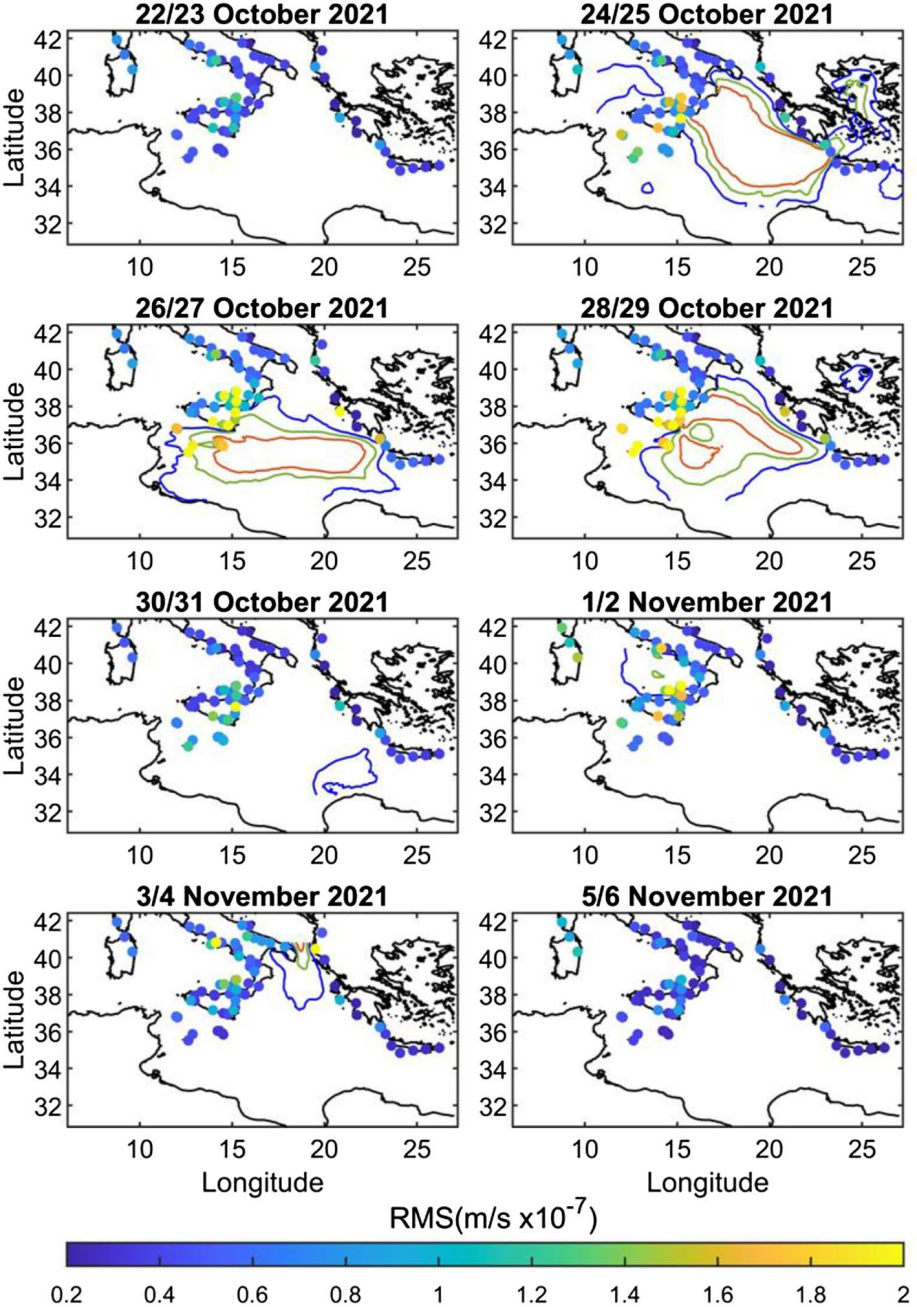
Spatial and temporal distribution of the RMS amplitude for the SM band. Each dot represents a station while the colors represent the RMS amplitude as specified in the color bar. The blue, green and orange contours line represent respectively significant wave heights of 2, 2.5 and 3 m obtained from the Copernicus product MEDSEA_HINDCAST_WAV_006_012.

To map the space–time distribution of the microseism amplitude we plotted the mean RMS amplitude computed on 2-day-long moving windows for the three different frequency bands (PM and SPSM in Supplementary Figs. 3, 4, SM Fig. 3). Also in these figures, we can note that in the PM (Supplementary Fig. 3) band the space–time distribution of the RMS amplitude is not related to Apollo, while in the SM (Fig. 3) and SPSM (Supplementary Fig. 4) bands we observe a spatial and temporal relationship between RMS amplitude and Apollo positions. In detail, in the map of the days 26–29 October (that represent the days when Apollo reached its maximum intensity) we can see a cluster of high values of RMS amplitude in the stations installed in the Ionian area (eastern Sicily, Sicily Channel and western Greece), highlighting a good match with the cyclone position.

If we compare our spectral analysis results with the results obtained in other works dealing with hurricanes, typhoons and tropical cyclones we can observe some similar features and other different characteristics. In the spectrograms shown in Lin et al.^28^, a trend similar to that of our spectrograms can be observed. In particular, in their study, it is possible to observe a rapid increase of the power spectral density in the SM and SPSM bands in correspondence with the development of the typhoon Megi, similarly to what we have obtained in our analyses (Fig. 2). Focusing on the intensity of the microseism signal, we can observe an increase in intensity in all the three microseism bands in Lin et al.^28^, while in the case of the Medicane Apollo, the increase in intensity takes place only in SM and SPSM bands. This difference could be due to the different sizes of Apollo and Megi, the diameter of a tropical cyclones (typhoons are a particular tropical cyclones which develop in northern Pacific Ocean and in Japan) is typically around 500 to 1000 km^33^, while a Medicane has a diameter on average of about 100–300 km^34^. Furthermore, the typhoon Megi has made the landfall, leading to the generation of PM as a consequence of the interaction between wave motions and ocean bottom in shallow waters, while Apollo always stays away from the coast and in an area with sea depth greater than 2000 m. As already described in the literature^20^, large water depths inhibit the generation of the PM as a consequence of the amplitude decay of pressure fluctuations that generate the signal, as a function of the depth. Specifically, the generation of the PM occurs only for depths less than ½ λ (where λ is the wavelength of the oceanic waves that generate the pressure fluctuations). Similar results are shown in Zhang et al.^31^. Also in this case the spectrograms show a rapid increase of the microseism amplitude for the SM and SPSM bands simultaneously with the development of the Ioke typhoon.

To track the movement of the Medicane Apollo we use two different complementary methods : (i) a grid search method based on seismic amplitude decay and (ii) array analysis. These methods are explained in the “Data and Methods” section.

**Figure 4:**
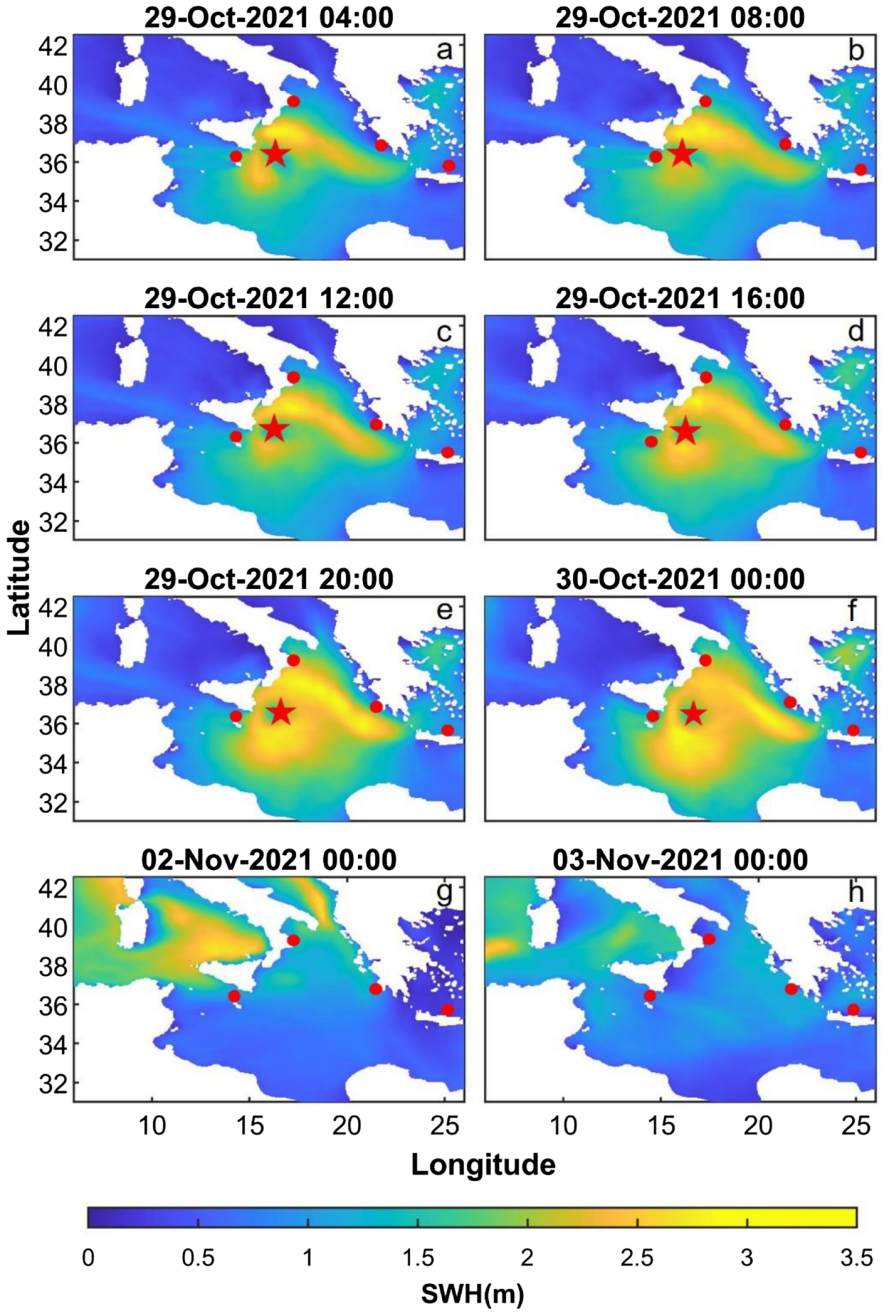
Hindcast maps showing the significant wave heights (﻿SWH; in m) during the period under investigation obtained from the Copernicus product MEDSEA_HINDCAST_WAV_006_012. In these images, Apollo and the cyclone eye (red star) are clearly visible. The red dots show the locations of the four oceanographic buoys used in this study.

**Figure 5:**
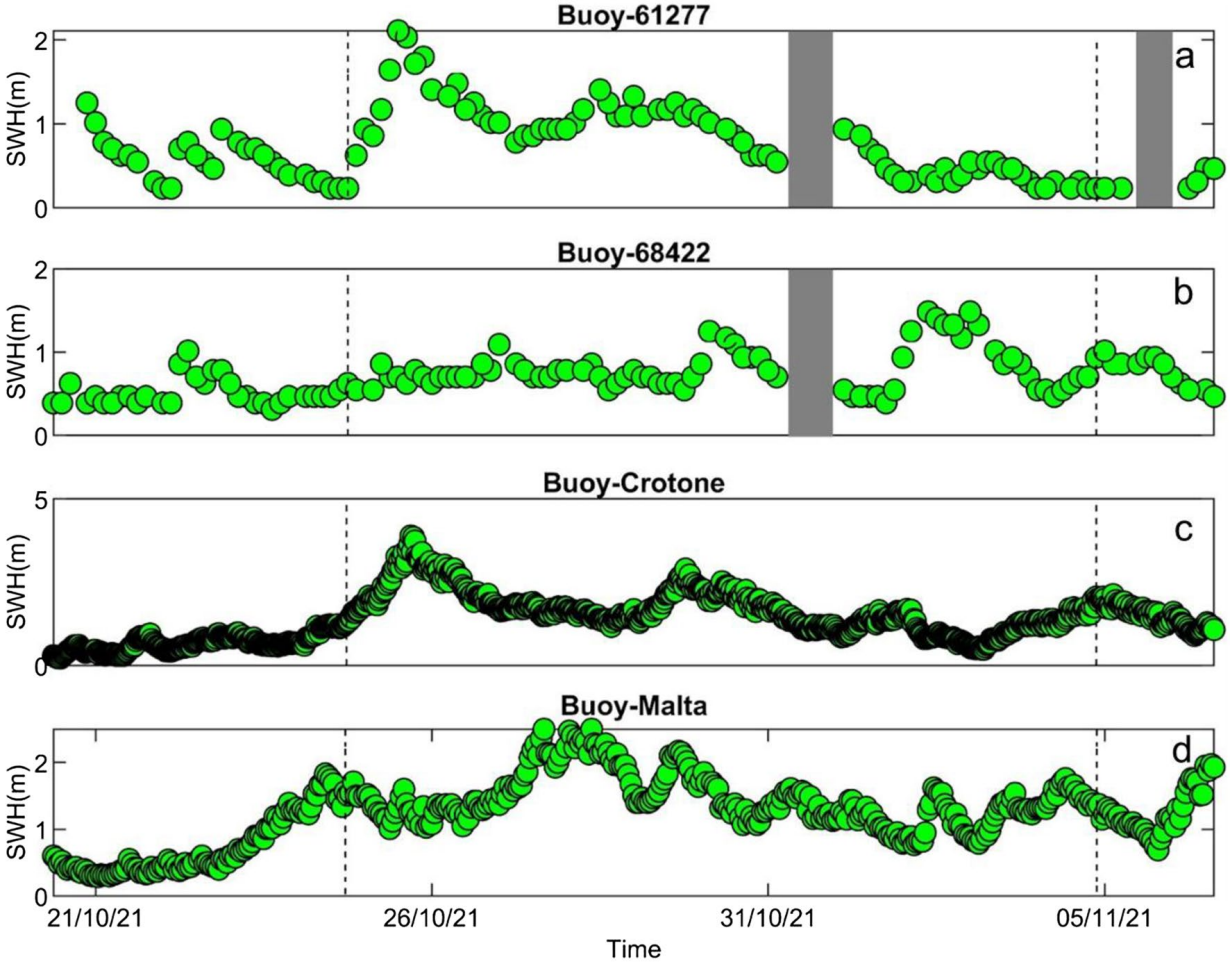
Significant wave heights (﻿SWH; in m) time series, recorded by the four buoys used in this study. The grey rectangles indicate a lack of data. The positions of the four buoys are shown in Fig. 1a.

**Figure 6:**
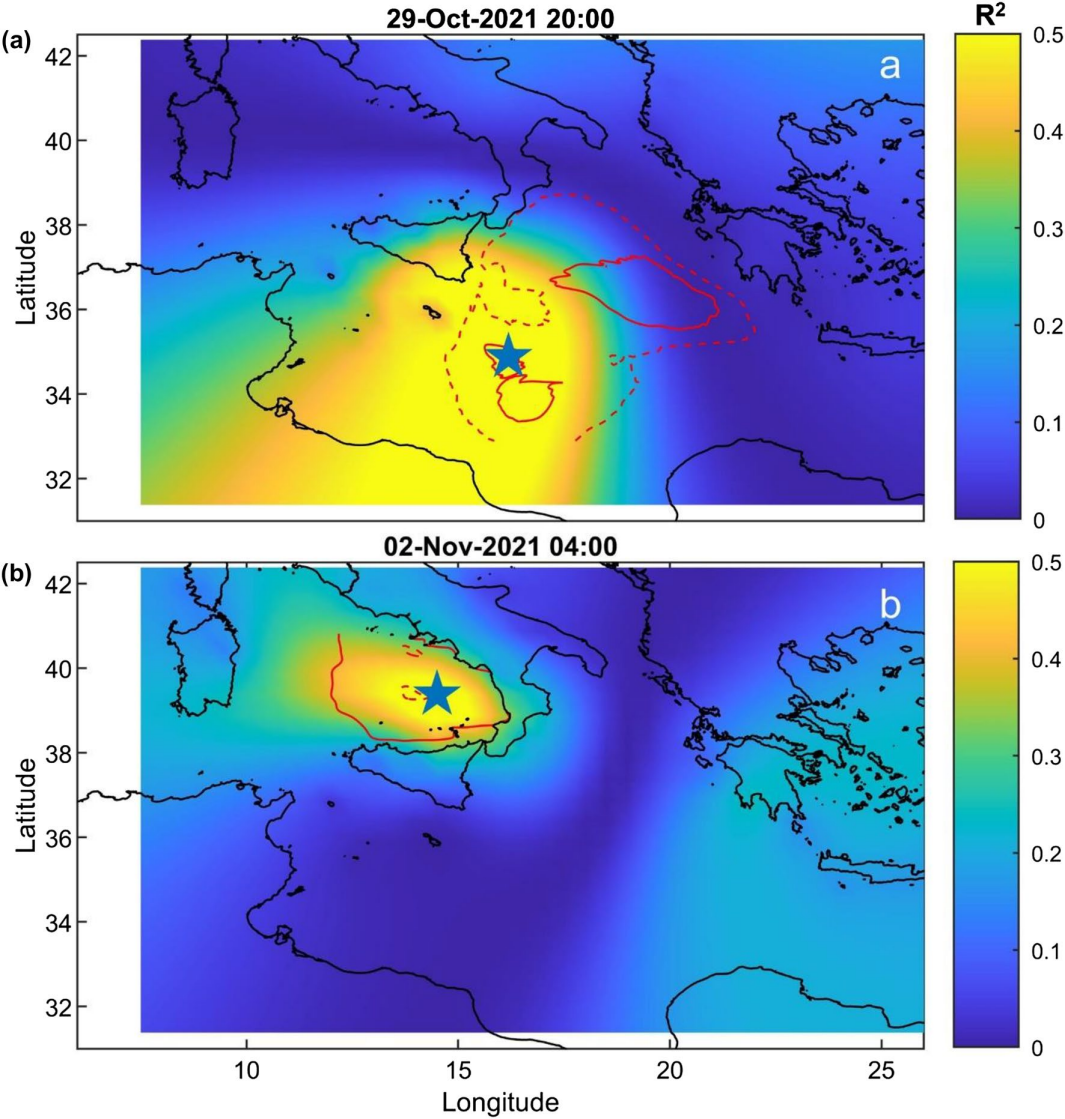
Localization of the microseism source for the period 29 October 2021 20:00 (**a**) and 2 November 2021 04:00 (**b**). The blue five-point star indicates the centroid position of all the grid nodes whose R^2^ values do not differ by more than 1% from the maximum R^2^ value. The red line represents the significant wave heights (in m) obtained from the Copernicus product MEDSEA_HINDCAST_WAV_006_012. In particular the dotted line represents significant wave heights of 2 m, the dashed line represents significant wave heights of 2.5 m and the solid line represents significant wave heights of 3 m.

With regard to the grid search method, we obtained the localization of the source regions in agreement with areas where significant wave heights greater than 3.5 m were observed (Figs. 4, 5c). In particular, we can consider two different time intervals: (i) 29–30 October 2021 (Figs. 4a–f, 6a) and ii) 2–3 November 2021 (Figs. 4g–h, 6b). We take into account only these two specific time intervals since only during these periods significant values of R^2^ (R^2^ > 0.5) are reached. During the former, representing the days when Apollo reached his climax, we obtained source locations in accord with the Medicane position in the Ionian Sea; in addition by performing one localization every four hours we were able to track the movements of Apollo. In particular, we obtained the first significant location on 29 October at 04:00. From this time we can follow the northward shift of Apollo until 29 October at 20:00 (Fig. 6a) while for the whole day of 30 October we are able to follow the southward motion of the Medicane (Supplementary Fig. 5). The results obtained with this method are consistent with the real shift of the Medicane. Indeed Apollo developed on 25 October near the Libyan coast, moving northward until 29 October and successively moving southeastward losing power. In the latter case, during 2–3 November 2021, we focused on a two-day-long interval (2–3 November) characterised by sustained winds and consequently violent sea wave motions in the Tyrrhenian area, near the Eolian Islands (Figs. 4g–h and 6b). Also in this case we were able to identify the position of the source region of the microseism, which, in accordance with the aforementioned weather data, was located in the Eolian area, keeping a stable position. In Table 1, the main features of the microseism source localised in these two specific time intervals are summarised. If we consider R^2^ values (Supplementary Fig. 6) we can observe that in the time interval 28–30 October 2021 we obtain the maximum values of the entire period, suggesting the clear predominance of the microseism source related to Apollo compared to the other sources. In particular the maximum R^2^ value is 0.65, obtained for the localization of 29 October 2021 at 12:00 AM, and is concordant with the time when Apollo reaches his maximum intensity. The linear regression of this localization is shown in the Supplementary Fig. 7.

**Table 1:** Main feature of the microseism source located in the two time intervals that show R^2^ values greater than 0.5.

Date	Hour	Longitude	Latitude	R^2^ value
29/10/2021	04:00	14.1053	33.3274	0.5936
29/10/2021	08:00	15.3846	33.8800	0.6474
29/10/2021	12:00	16.1250	34.6300	0.6533
29/10/2021	16:00	16.2143	34.7371	0.6138
29/10/2021	20:00	16.1875	34.8800	0.5654
30/10/2021	00:00	16.2500	33.8800	0.5380
02/11/2021	08:00	13.5000	39.8800	0.5667
02/11/2021	12:00	13.5000	39.8800	0.5887
02/11/2021	16:00	14.2500	39.8800	0.5799
02/11/2021	20:00	14.500	39.8800	0.5589
03/11/2021	00:00	13.7500	39.0467	0.5028

Although on 2–3 November Apollo was still present, we can optimally localise the wind storm in the Tyrrhenian area since this storm showed greater intensity than Apollo during the beginning of November and for this reason, it became the predominant source compared to the Medicane.

For these two specific time intervals, we also estimated the mean value of the exponent *b* (derived from the geometrical spreading, microseism amplitude was considered proportional to *r*^−*b*^, where *r* is the source—station distance and the exponent *b* should be equal to 1 or 0.5 in the case of body or surface waves, respectively) and we obtained a value close to 1. This result is apparently in contrast with the predominance of surface waves in the microseism wavefield^16, 35, 36^. Not excluding a contribution of body waves in the microseism wavefield, it can be explained also considering an “excessive” amplitude difference between the stations installed closer to the Medicane Apollo and those further away which results in a greater inclination of the straight-line fitting seismic amplitude versus source-station distance in the logarithmic graph. Indeed, it is likely that the stations, installed onshore in the area closer to the Medicane Apollo, are influenced by local seismic sources such as wind or rain. These local sources contribute to increase the amplitude recorded at these stations and lead to an overestimation of the seismic amplitude.

It is worth noting that the locations of the microseism source, obtained in the interval 28–30 October, do not match exactly with the cyclone eye but are located slightly southward. This could be due to the fact that the generation of the SM depends on the interaction between two different wave trains with similar features but travelling in opposite directions^14, 17–19^. The coexistence of two wave trains in opposite directions is not likely to occur in the cyclone's eye but in the outermost regions of the cyclone. In particular, in these areas, there could be wave trains generated from the cyclone that interact with “external” wave trains or with other waves previously generated by the cyclone^29^.

**Figure 7:**
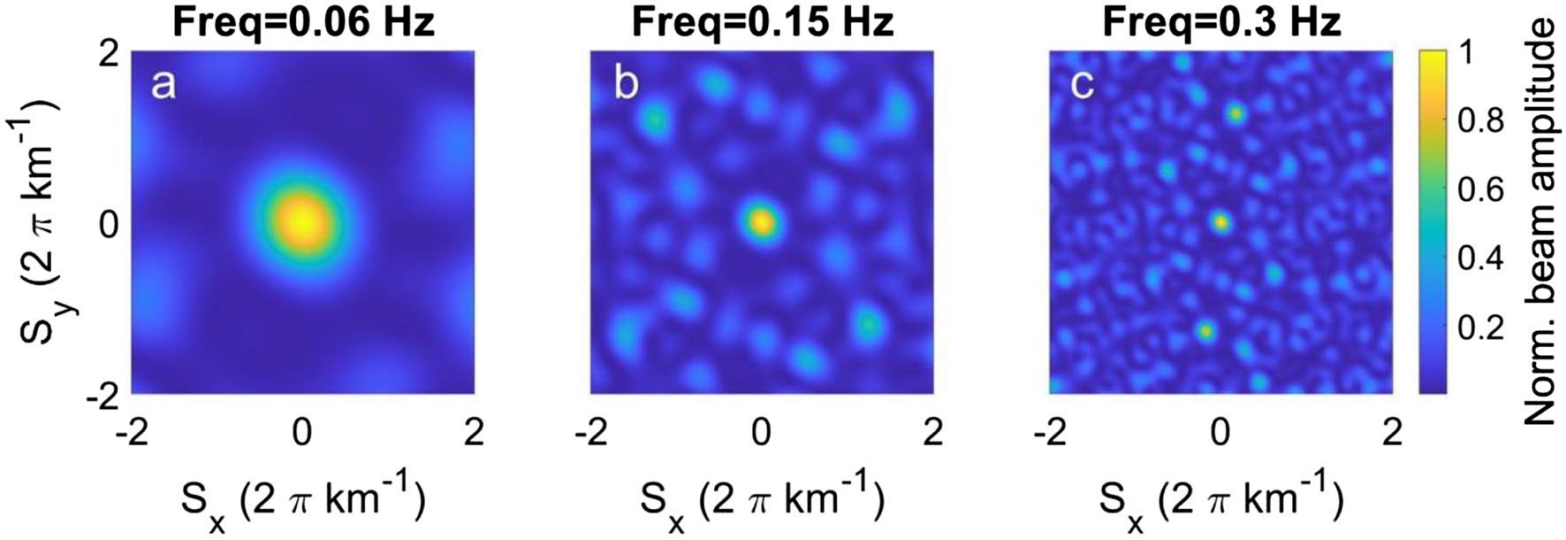
Array response functions of the fifteen stations composing the Mt. Etna seismic permanent network (see Fig. 1b) for a unit amplitude incident wave with slowness of 0 s/km at frequency of PM (**a**), SM (**b**), and SPSM (**c**).

**Figure 8:**
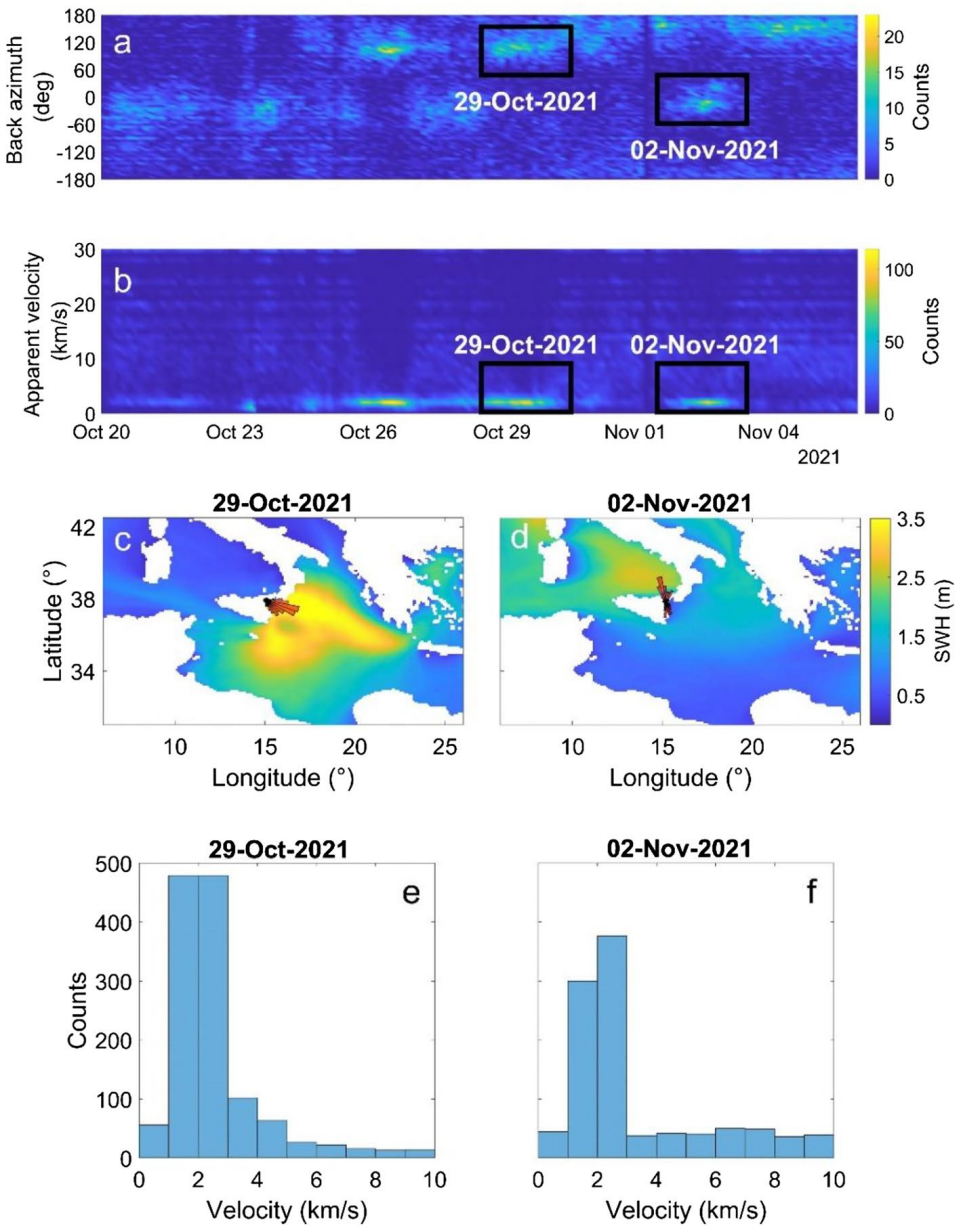
Distribution of back azimuth and apparent velocity values computed by f-k analysis for the SM frequency band. (**a**) Temporal histogram of back azimuth values, (**b**) Temporal histogram of apparent velocity values, (**c**, **d**) Hindcast maps of the significant wave heights (SWH; in m) with rose diagrams, located at the centre of the summit area of Mt. Etna (see Fig. 1b), showing the distribution of the back azimuth values on 29/10/2021 and 02/11/2021, respectively, (**e**, **f**) Histograms of apparent velocity values for 29/10/2021 and 02/11/2021, respectively.

Concerning the array analysis, the Mt. Etna seismic permanent network turned out to be a reliable array to locate the microseism sources in the SM band (Fig. 7b). Indeed, during the days when maximum Apollo intensity was observed, back azimuth values pointed toward the cyclone position with apparent velocity values of ~ 1.0–3.0 km/s (Fig. 8a,b). When Apollo activity involves the Ionian coastlines (Fig. 4a–f), back azimuth values indicate the Catania Gulf, pointing south-eastward (Fig. 8c). Alternatively, when the highest significant wave heights are observed in the Tyrrhenian Sea (Fig. 4g,h), the back azimuth values rotate pointing north-westward (Fig. 8e).

The results of array analysis are in agreement with some studies demonstrating that the back azimuth and apparent velocity of microseism are well-correlated to ocean-wave heights^37^ or hurricanes/cyclones^26^. In particular, the time series of back azimuth highlight a good match between SM source and Apollo positions (Fig. 8a,c,e), as reported in other studies concerning the relationship between microseism sources and cyclonic activity^20, 28, 29, 31^. These results are also compatible with Moschella et al.^24^, who explored the microseism recorded along the coastline of Eastern Sicily. Although they focused on a different frequency band (SPSM), they showed through array analysis how the SPSM sources are located in the shallow waters of the Catania Gulf and the Northern Sicily coastlines during periods when the highest significant wave heights are observed in the Ionian Sea and the Tyrrhenian Sea, respectively. Concerning the seismic apparent velocity estimated in the SM band (Fig. 8b,d,f), the values of ~ 1.0–3.0 km/s are in agreement with the Rayleigh wave velocity calculated in Moschella et al.^24^, as well as with the wave velocity values retrieved by investigating the ambient seismic noise in the coastlines of the northeast of the Netherlands^38^, in the New Zealand^39^ and in the Valley of Mexico^40^.

The two applied location methods (grid search method and array analysis) can be considered complementary allowing us to compare the results obtained independently.

## Conclusions

Several studies have explored the relationship between microseism and cyclonic activity focusing on the link between secondary microseism (SM), short period secondary microseism (SPSM) bands and hurricanes, tropical cyclones and typhoons. In this work, we analysed the relationship between microseism, specifically in the SM band, and MEDIterranean hurriCANES, or Medicanes, never analysed before. For this reason, we took into account the Medicane Apollo, which occurred in the Ionian Sea between 25 October and 5 November 2021, and we selected 93 seismic stations (78 used in spectral and amplitude analysis and in the grid search method and 15 in array analysis) to reconstruct the microseism signature of these extreme Mediterranean weather events and to seismically track the position of Apollo during its lifetime.

From our spectral analyses, it is clear that the seismic signals, in the bands of SM and SPSM (0.1–0.2 and 0.2–0.4 Hz, respectively), are affected by the presence of Apollo, while the PM (0.05–0.07 Hz) band shows no significant variation. The absence of a relationship between Apollo and the PM band could be due both to the Apollo small-size, as well as to the fact that Apollo always remains away from the coastline, in areas where the depth (about 2000 m) is greater than ½λ (where λ is the wavelength of the oceanic waves that generate the pressure fluctuations). Indeed, as already described in the literature, high depths inhibit the generation of the PM as a consequence of the amplitude decay of pressure fluctuations.

Focusing on spectrograms, RMS amplitude time series and space–time distribution, we observe that the intensity of the microseism signals is greater in the time interval 25 October–5 November 2021. In addition, the stations showing the highest microseism amplitude are located near the Ionian coastline (for example EPOZ, FSK and PFKS), while the stations installed in the Tyrrhenian area show a different trend, probably caused by other seismic sources.

We could identify and track the position of the Medicane by two different methods: i) grid search method based on seismic amplitude decay and ii) array analysis. Indeed, the location results are in agreement with the real position of the Medicane and allowed us to follow the movement of Apollo when it reached its climax (28–30 October 2021).

This work represents the first approach to studying and monitoring these extreme Mediterranean weather events through the use of microseism. It shows how also the seismic data can play an important role in the development of an innovative system of monitoring of sea state integrating different kinds of instruments such as wavemeter buoy, radar HF, geostationary satellite and seismometers. In particular, the latter could compensate for the lack of data from the wavemeter buoys, more often affected by instrument breakage.

Finally, as seismometers are amongst the first geophysical instruments to be installed, microseism could allow us to analyse past extreme weather events in a way to compare the intensity of these meteorological phenomena in the climate change scenarios.

## Data and methods

### Seismic data

We selected 78 seismic stations (Fig. 1a) installed along Italian coastal areas (from Central Italy southward), along Greece coastal areas and in the Sicilian Channel (Linosa, Pantelleria and Malta) (Supplementary Table 1). In addition, to perform array analysis, 15 stations installed in the Etnean area (Fig. 1b) were used (Supplementary Table 2). The selected seismic stations exhibit specific characteristics: they are (i) installed in coastal areas and (ii) equipped with 3-component broadband seismic sensors.

### Sea state data

To obtain information about the sea state, the “MEDSEA_HINDCAST_WAV_006_012” product, produced by the Copernicus Marine Environment Monitoring Service, along with the significant wave height measured by four oceanographic buoys were used (Fig. 1a).

Concerning the former, the product contains the hindcast maps of the Mediterranean Sea Waves forecasting system and is based on the third-generation wave model WAM Cycle 4.5.4^41^. In particular, the hourly significant wave height data was used to reconstruct the sea wave state during Apollo Medicane (Fig. 2).

As for the latter, Fig. 1 reports the locations of four directional wave buoys. Two directional wave buoys (WMO code 68422 and 61277) are located along the Greek coastline, one buoy, Crotone^42^, is located in southern Italy and the last (Malta) lies in front of the northern part of Gozo Island.

For the analysed period, significant wave height data are shown in Fig. 5. Figure 5a, representing significant wave height for the 68422 buoy, shows that the Medicane was not observed by this point, nor by the 61277 buoy in terms of significant wave heights (Fig. 5b), although its temporal pattern is similar to the one of Crotone buoy where the increase of significant wave heights due to the Medicane passage is clearly visible (Fig. 5c). In particular, at the Crotone Buoy, the significant wave height reached the value of 3 m at 18:30 of 29 October 2021 with a SE mean direction, a peak period of 8.83 s, a mean period of 7.72 s and a value of barometric pressure equal to 1016.2 hPa. Also the maltese-buoy shows an increase of the significant wave heights in correspondence of the considered time interval (Fig. 5d). In particular, at the Malta Buoy the significant wave height reached the value of 2.50 m at 09:00 of 28 October 2021 with a south-eastern mean direction, a peak period of 7 s and a mean period of 5.85 s.

### Spectral analysis

The seismic data were corrected for the instrument response and successively spectral and amplitude analyses were performed. As for the former, hourly spectra of the seismic signal were calculated by using Welch’s method^43^ with windows of 81.92 s. All the hourly spectra were gathered and visualized as spectrograms, with time on the x axis, frequency on the y axis and the log_10_ of the power spectral density (PSD) indicated by a colour scale. Spectrograms obtained for the vertical component of four stations are shown in Fig. 2 as examples. Concerning the amplitude, we calculated hourly RMS (root mean square) amplitude time series for the following frequency bands: 0.2–0.4 Hz (SPSM, Supplementary Fig. 1), 0.1–0.2 Hz (SM, Fig. 2) and 0.05–0.07 Hz (PM, Supplementary Fig. 2).

In order to show the spatial and temporal distribution of the RMS amplitude during the period under investigation, we plotted the mean RMS amplitude computed on 2-day-long moving windows for the three different frequency bands (Fig. 3 and Supplementary Figs. 3, 4). Each dot represents a seismic station and the colour of the dot relates to the corresponding RMS amplitude at that location, as specified in the color bar. Noteworthy, each color bar shows a different range of RMS amplitude highlighting a different response of the three considered microseism frequency bands (PM, SM and SPSM) to the cyclonic activity (Fig. 3 and Supplementary Figs. 3, 4).

### Tracking Apollo position

Since the microseism signal is continuous, it is impossible to apply the conventional techniques based on the first phase arrivals used to localise the hypocenter of a seismic event. For this reason, we applied two different methods: (i) grid search method, based on seismic amplitude-decay, and (ii) array analysis.

### Grid search method

We used the seismic signals recorded by the 78 aforementioned stations (Fig. 1a) with the aim of tracking the position of the Medicane Apollo during its lifetime by using a grid search approach (Fig. 6). This method, based on seismic amplitude decay, has already been used to track the source of the volcanic tremor at Mt. Etna^44, 45^. In this method, we make the assumption that the seismic waves are propagating inside a homogeneous medium (for further details about the method see Cannata et al.^46^). Furthermore, due to the geometrical spreading, microseism amplitude was considered proportional to *r*^−*b*^, where *r* is the source—station distance and the exponent *b* should be equal to 1 or 0.5 in the case of body or surface waves, respectively. The latter parameter was left unconstrained in the location procedure. To take into account also intrinsic attenuation, the frequency-dependent absorption coefficient α was considered in the range 0–0.25 × 10^–3^ km^−1^^47^. As the sources of microseism are located on the solid Earth's surface, the search was carried out on a planar 2D grid roughly coinciding with the Earth's surface. This is different from what is performed on volcanoes where a 3D grid search is carried out to locate also deep seismic sources. Concerning the region where we executed the grid search, it has an area of 1716 km × 1366 km (maximum longitude: 29°; minimum longitude: 9°; maximum latitude: 42.48°; minimum latitude: 30.18°) with a spacing of 0.05°. As shown by Cannata et al.^46^ and by other authors applying similar grid-search-based methods (for example Battaglia et Aki^48^ used this method to localize eruption tremor sources on the Piton de la Fournaise volcano, or Kumagai et al.^49^ applied this approach to localize the ascending seismic source during an explosive eruption at Tungurahua volcano), the grid spacing is chosen as a compromise between good spatial resolution and reasonable computation time. The microseism source is localized on the basis of the goodness of the linear regression fit (hereafter referred to as R^2^) obtained for each node of the bidimensional (2D) grid previously mentioned. Specifically, the source was located in the centroid position of all the grid nodes whose R^2^ values do not differ by more than 1% from the maximum R^2^ value. In order to assess the reliability of the location results, we applied a method to evaluate the statistical significance of the retrieved maximum R^2^ value. In particular, we performed 2142 runs by randomly shuffling the RMS amplitude values among the stations. Then, we calculated the 99th percentile of the 2142 values of R^2^ and obtained 0.49. Hence, we only considered reliable the source locations with R^2^ values greater than 0.49 (Supplementary Fig. 8).

The considered method shows various limits that in particular cases can significantly affect the source locations. In particular, the first limit concerns the existence of multiple sources that exhibit similar intensity within the same frequency range. In that case, the constrained source location shifts toward a position in between the real seismic source locations^48^ and the R^2^ dramatically decreases. In our case, we neglect localization that shows R^2^ values smaller than 0.49 in order to avoid such a problem. Another limit to take into account concerns the fact that in this method we consider the microseism source as a point-like source, while the microseism, taken into account in this case study, integrates over a wide area of the Mediterranean Sea. In this case the point-like source is localized as the barycentric point of the extended source.

### Array analysis

In order to track the location of Apollo Medicane, fifteen stations belonging to the Mt. Etna seismic permanent network were used as a roughly circular array (Fig. 1b). The array analysis was carried out to measure the apparent velocity and back azimuth of the arriving microseism signal^50^. Most of the array techniques assume a planar propagation of the wavefront across the array on the basis of the relationship between the sensor-source distances and the wavelength of the signal of interest^51^. The resolution of the array analysis depends on the geometry/size of the array and the wavelength of the seismic signal^51^. Three conditions have to be met with respect to the array configuration: (i) the aperture of the array should be greater than a quarter of the signal wavelength that we want to analyse^52^; (ii) to avoid spatial aliasing, the wavelength of the signal should be at least comparable with the array interspacing^53^; (iii) distances between array receivers and source of the signal must be greater than one wavelength^51^.

Array Response function (ARF) is a good tool to plan the array geometry required to investigate microseism signals. The ARFs were computed for the PM, SM and SPSM frequency bands by using the Beam pattern function^54^ for a vertically incident plane wave (Fig. 7). Such ARFs exhibit that the roughly circular array has a good response only for the SM case (Fig. 7b). In fact, considering a velocity of the S-waves (Vs) in the first kilometres of the crust equal to ~ 2 km/s^55^, the wavelengths of SM (10–20 km) are comparable with the aperture (~ 16 km) and interspacing of the array (~ 6 km), in accordance with the conditions (i) and (ii) mentioned in the previous paragraph. In addition, supposing that the microseism sources are located at minimum distances of ~ 20 and 45 km from the centre of the array (distances from the Ionian Sea and the Tyrrhenian Sea, respectively), the Etna circular array should be able to locate the microseism sources with a planar wavefront assumption (condition (iii)).

In this work, we used the f-k (frequency-wavenumber) analysis on microseism signals^50^. This method consists of a beamforming approach in the spectral domain, seeking in a grid search of slowness the back azimuth and apparent velocity values for which the amplitude of the sum of all the array traces is maximised. The result of the f-k analysis is power spectral density as a function of slowness. To apply array analysis on microseism, the following processing steps were carried out on the seismic signals: (i) demeaning and detrending; (ii) filtering within a specific frequency band of microseism; (iii) subdivision in 60-s-long tapered windows; (iv) excluding windows with seismo-volcanic amplitude transients (i.e. volcano-tectonic earthquakes, long-period events, very long period events) detected by using STA/LTA technique^56^; (v) applying f-k analysis for each window by using a slowness grid search (from − 1 to 1 s/km in the east and north components of the slowness vector) with a spacing of 0.05 s/km. An example of the results is shown in Fig. 8.

## Supplementary Information

Supplementary Figures.

Supplementary Table 1.

Supplementary Table 2.

## Data Availability

The seismic data of the Italian, Greek and some Maltese stations used in this work are available on the Orfeus website (http://www.orfeus-eu.org/). The data of the others Maltese seismic stations, not available on Orfeus website, are available by contacting the Seismic Research Group within the Department of Geoscience of the University of Malta (smru-web@um.edu.mt). The Greek buoys (WMO code 68422 and 61277) data are available on the Copernicus European project website (https://www.copernicus.eu/en), the Italian buoy data (Crotone) on the ISPRA website (http://dati.isprambiente.it/dataset/ron-rete-ondametrica-nazionale/) and the Maltese buoy data on the website of the Physical Oceanography Research Group of the Malta university (http://ioi.research.um.edu.mt/news-waves/). The hindcast data (“MEDSEA_HINDCAST_WAV_006_012” product) are available on the Copernicus European project website (https://www.copernicus.eu/en).

## References

[1] Faranda D (2022). A climate-change attribution retrospective of some impactful weather extremes of 2021. Weather Clim. Dyn. Discuss..

[2] Miglietta MM, Rotunno R (2019). Development mechanisms for Mediterranean tropical-like cyclones (medicanes). Q. J. R. Meteorol. Soc..

[3] Miglietta MM (2011). Numerical analysis of a Mediterranean ‘hurricane’ over south-eastern Italy: Sensitivity experiments to sea surface temperature. Atmos. Res..

[4] Miglietta MM (2013). Analysis of tropical-like cyclones over the Mediterranean Sea through a combined modeling and satellite approach. Geophys. Res. Lett..

[5] Cavicchia L, von Storch H, Gualdi S (2014). A long-term climatology of medicanes. Clim. Dyn..

[6] Nastos PT, KaravanaPapadimou K, Matsangouras IT (2018). Mediterranean tropical-like cyclones: Impacts and composite daily means and anomalies of synoptic patterns. Atmos. Res..

[7] Ivan R, Oliver J, Mia F, Renato F (2018). A study of GPS positioning error associated with tropospheric delay during Numa Mediterranean cyclone. Int. J. Traffic aTransp. Eng..

[8] Portmann R, González-Alemán JJ, Sprenger M, Wernli H (2020). How an uncertain short-wave perturbation on the North Atlantic wave guide affects the forecast of an intense Mediterranean cyclone (Medicane Zorbas). Weather Clim. Dyn..

[9] Scicchitano G (2020). The first video witness of coastal boulder displacements recorded during the impact of medicane ‘Zorbas’ on Southeastern Sicily. Water.

[10] Varlas G (2020). Investigating the impact of atmosphere–wave–ocean interactions on a Mediterranean tropical-like cyclone. Ocean Model.

[11] Lagouvardos K, Karagiannidis A, Dafis S, Kalimeris A, Kotroni V (2022). Ianos: A hurricane in the Mediterranean. Bull. Am. Meteor. Soc..

[12] Zimbo F, Ingemi D, Guidi G (2022). The tropical-like cyclone “ianos” in September 2020. Meteorology.

[13] Gazzetta Ufficiale Della Repubblica Italiana. https://www.gazzettaufficiale.it/eli/gu/2022/01/22/17/sg/pdf.

[14] Longuet-Higgins MS (1950). A theory of the origin of microseisms. Philos. Trans. R. Soc. Lond. A.

[15] Hasselmann K (1963). A statistical analysis of the generation of microseisms. Rev. Geophys..

[16] Haubrich RA, McCamy K (1969). Microseisms: Coastal and pelagic sources. Rev. Geophys..

[17] Ardhuin F, Gualtieri L, Stutzmann E (2015). How ocean waves rock the Earth: Two mechanisms explain microseisms with periods 3 to 300s. Geophys. Res. Lett..

[18] Ardhuin F, Balanche A, Stutzmann E, Obrebski M (2012). From seismic noise to ocean wave parameters: General methods and validation. J. Geophys. Res. Oceans.

[19] Oliver J, Page R (1963). Concurrent storms of long and ultralong period microseisms. Bull. Seismol. Soc. Am..

[20] Bromirski PD, Duennebier FK, Stephen RA (2005). Mid-ocean microseisms. Geochem. Geophys. Geosyst..

[21] Ardhuin F (2019). Observing sea states. Front. Mar. Sci..

[22] Cannata A (2020). Unravelling the relationship between microseisms and spatial distribution of sea wave height by statistical and machine learning approaches. Remote Sens..

[23] Guerin G (2022). Quantifying microseismic noise generation from coastal reflection of gravity waves recorded by seafloor DAS. Geophys. J. Int..

[24] Moschella S (2020). Insights into microseism sources by array and machine learning techniques: Ionian and Tyrrhenian sea case of study. Front. Earth Sci..

[25] Bromirski PD (2001). Vibrations from the “perfect storm”. Geochem. Geophys. Geosyst..

[26] Gerstoft P, Fehler MC, Sabra KG (2006). When Katrina hit California. Geophys. Res. Lett..

[27] Gualtieri L, Camargo SJ, Pascale S, Pons FME, Ekström G (2018). The persistent signature of tropical cyclones in ambient seismic noise. Earth Planet. Sci. Lett..

[28] Lin J, Lin J, Xu M (2017). Microseisms generated by super Typhoon Megi in the Western Pacific Ocean. J. Geophys. Res. Oceans.

[29] Retailleau L, Gualtieri L (2019). Toward high-resolution period-dependent seismic monitoring of tropical cyclones. Geophys. Res. Lett..

[30] Retailleau L, Gualtieri L (2021). Multi-phase seismic source imprint of tropical cyclones. Nat. Commun..

[31] Zhang J, Gerstoft P, Bromirski PD (2010). Pelagic and coastal sources of P-wave microseisms: Generation under tropical cyclones. Geophys. Res. Lett..

[32] Lecocq T, Ardhuin F, Collin F, Camelbeeck T (2020). On the extraction of microseismic ground motion from analog seismograms for the validation of ocean-climate models. Seismol. Res. Lett..

[33] Chan JCL (2005). The physics of tropical cyclone motion. Annu. Rev. Fluid Mech..

[34] Prat AC (2021). Evaluation of the sensitivity of medicane ianos to model microphysics and initial conditions using satellite measurements. Remote Sens..

[35] Bromirski PD (2002). The near-coastal microseism spectrum: Spatial and temporal wave climate relationships. J. Geophys. Res. Solid Earth.

[36] Gualtieri L, Bachmann E, Simons FJ, Tromp J, Romanowicz BA (2020). The origin of secondary microseism Love waves. Proc. Natl. Acad. Sci..

[37] Chevrot S (2007). Source locations of secondary microseisms in western Europe: Evidence for both coastal and pelagic sources. J. Geophys. Res. Solid Earth.

[38] Kimman WP, Campman X, Trampert J (2012). Characteristics of seismic noise: Fundamental and higher mode energy observed in the northeast of the Netherlands. Bull. Seismol. Soc. Am..

[39] Brooks LA, Townend J, Gerstoft P, Bannister S, Carter L (2009). Fundamental and higher-mode Rayleigh wave characteristics of ambient seismic noise in New Zealand. Geophys. Res. Lett..

[40] Rivet D, Campillo M, Sanchez-Sesma F, Shapiro NM, Singh SK (2015). Identification of surface wave higher modes using a methodology based on seismic noise and coda waves. Geophys. J. Int..

[41] Kieser, J. *et al*. First studies with the high-resolution coupled wave current model CWAM and other aspects of the project Sea State Monitor. in *Proceedings of the 13th International Workshop on Wave Hindcasting and 4th Coastal Hazard Symposium, Presentation. Banff, Canada*. (2013). http://www.waveworkshop.org/13thWaves/index.htm Accessed 27 Oct 2015.

[42] Bencivenga M, Nardone G, Ruggiero F, Calore D (2012). The Italian data buoy network (RON). Adv. Fluid Mech. IX.

[43] Welch PD (1967). The use of fast fourier transform for the estimation of power spectra: A method based on time averaging over short, modified periodograms. IEEE Trans. Audio Electroacoust..

[44] Di Grazia G, Falsaperla S, Langer H (2006). Volcanic tremor location during the 2004 Mount Etna lava effusion. Geophys. Res. Lett..

[45] Cannata A (2010). New insights into banded tremor from the 2008–2009 Mount Etna eruption. J. Geophys. Res. Solid Earth.

[46] Cannata A (2013). Monitoring seismo-volcanic and infrasonic signals at volcanoes: Mt Etna case study. Pure Appl. Geophys..

[47] Mitchell BJ (1995). Anelastic structure and evolution of the continental crust and upper mantle from seismic surface wave attenuation. Rev. Geophys..

[48] Battaglia J, Aki K, Ferrazzini V (2005). Location of tremor sources and estimation of lava output using tremor source amplitude on the Piton de la Fournaise volcano: 1. Location of tremor sources. J. Volcanol. Geotherm. Res..

[49] Kumagai H, Placios P, Ruiz M, Yepes H, Kozono T (2011). Ascending seismic source during an explosive eruption at Tungurahua volcano, Ecuador. Geophys. Res. Lett..

[50] Rost S, Thomas C (2002). Array seismology: Methods and applications. Rev. Geophys..

[51] Havskov J, Alguacil G (2015). Instrumentation in earthquake seismology. Instrum. Earthq. Seismol..

[52] Aster RC, Scott J (1993). Comprehensive characterization of waveform similarity in microearthquake data sets. Bull. Seismol. Soc. Am..

[53] Asten MW, Henstridge JD (1984). Array estimators and the use of microseisms for reconnaissance of sedimentary basins. Geophysics.

[54] Capon J (1969). High-resolution frequency-wavenumber spectrum analysis. Proc. IEEE.

[55] Patanè D, Ferrucci F, Gresta S (1994). Spectral features of microearthquakes in volcanic areas: Attenuation in the crust and amplitude response of the site at Mt Etna, Italy. Bull. Seismol. Soc. Am..

[56] Trnkoczy, A. *Understanding and Parameter Setting of STA/LTA Trigger Algorithm. In New Manual of Seismological Observatory Practice (NMSOP)*, 1–20. (Deutsches GeoForschungsZentrum GFZ, 1999).

