# Supplementary material for: Monitoring extreme meteo-marine events in the Mediterranean area using the microseism (Medicane Apollo case study)
Source: Sci Rep. 2022 Dec 9;12:21363. doi: 10.1038/s41598-022-25395-9 (PMC9734140; doi:10.1038/s41598-022-25395-9)
Supplement: Supplementary file 1 — Supplementary Figures. [file 41598_2022_25395_MOESM1_ESM.docx]

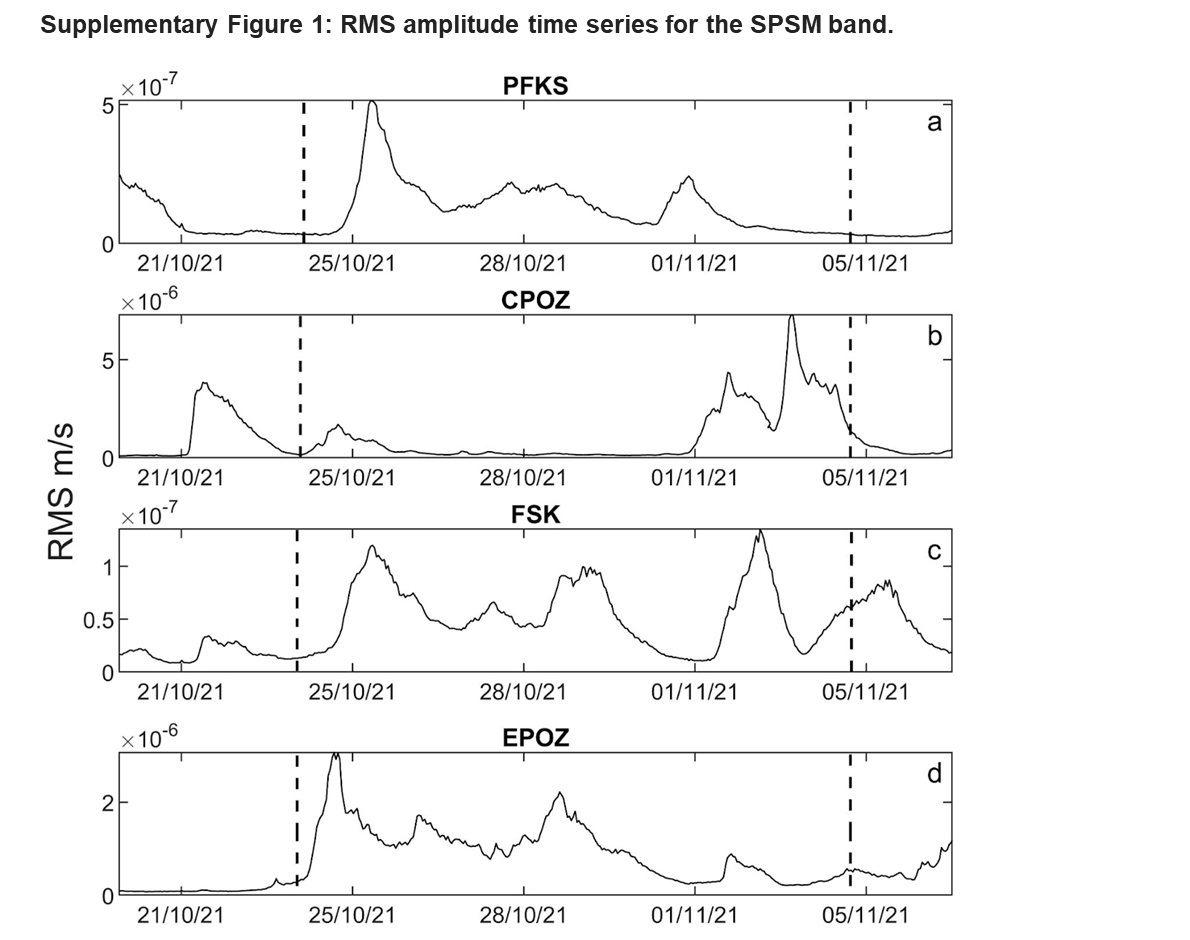


**Supplementary Figure 1.** RMS amplitude time series, for the SPSM band, of the seismic signal recorded by the vertical component of 4 considered stations located along the Greek coastline (a and c), in central Italy (b) and in the eastern part of Sicily (d) (see **Figure 1a** for the station locations).


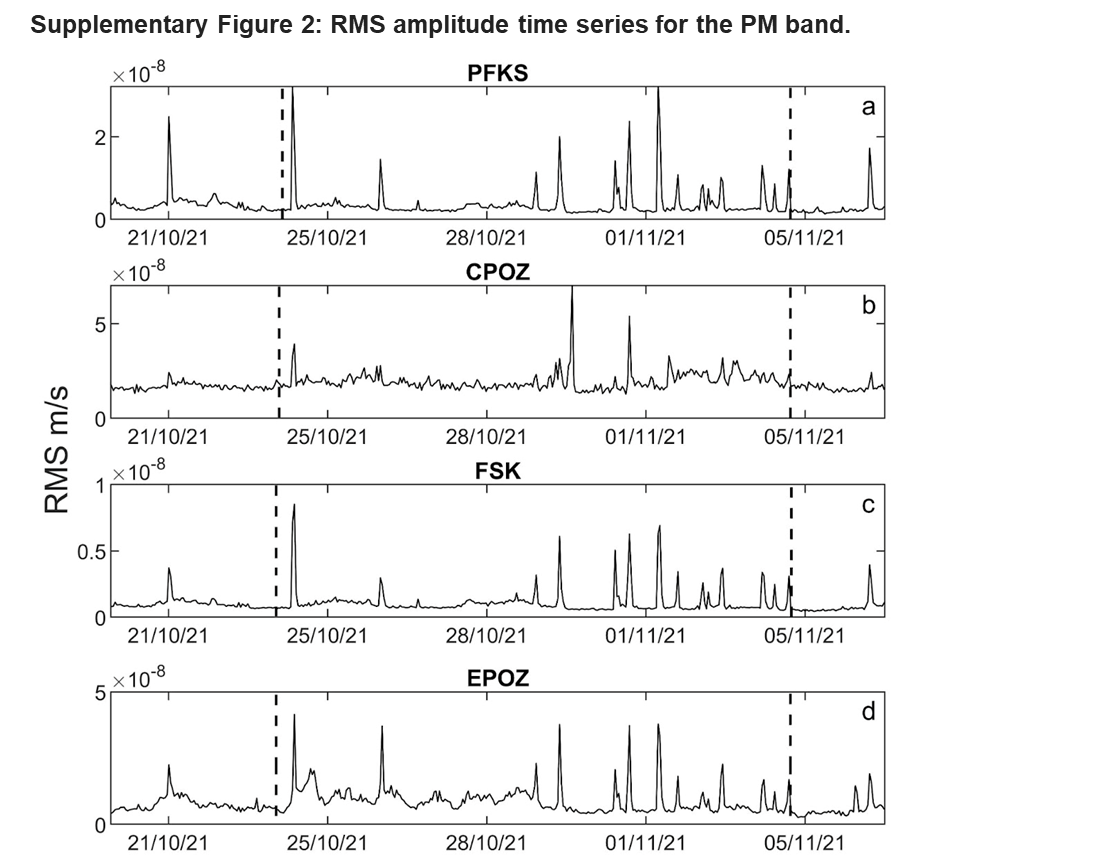


**Supplementary Figure 2.** RMS amplitude time series, for the PM band, of the seismic signal recorded by the vertical component of 4 considered stations located along the Greek coastline (a and c), in central Italy (b) and in the eastern part of Sicily (d) (see **Figure 1a** for the station locations).


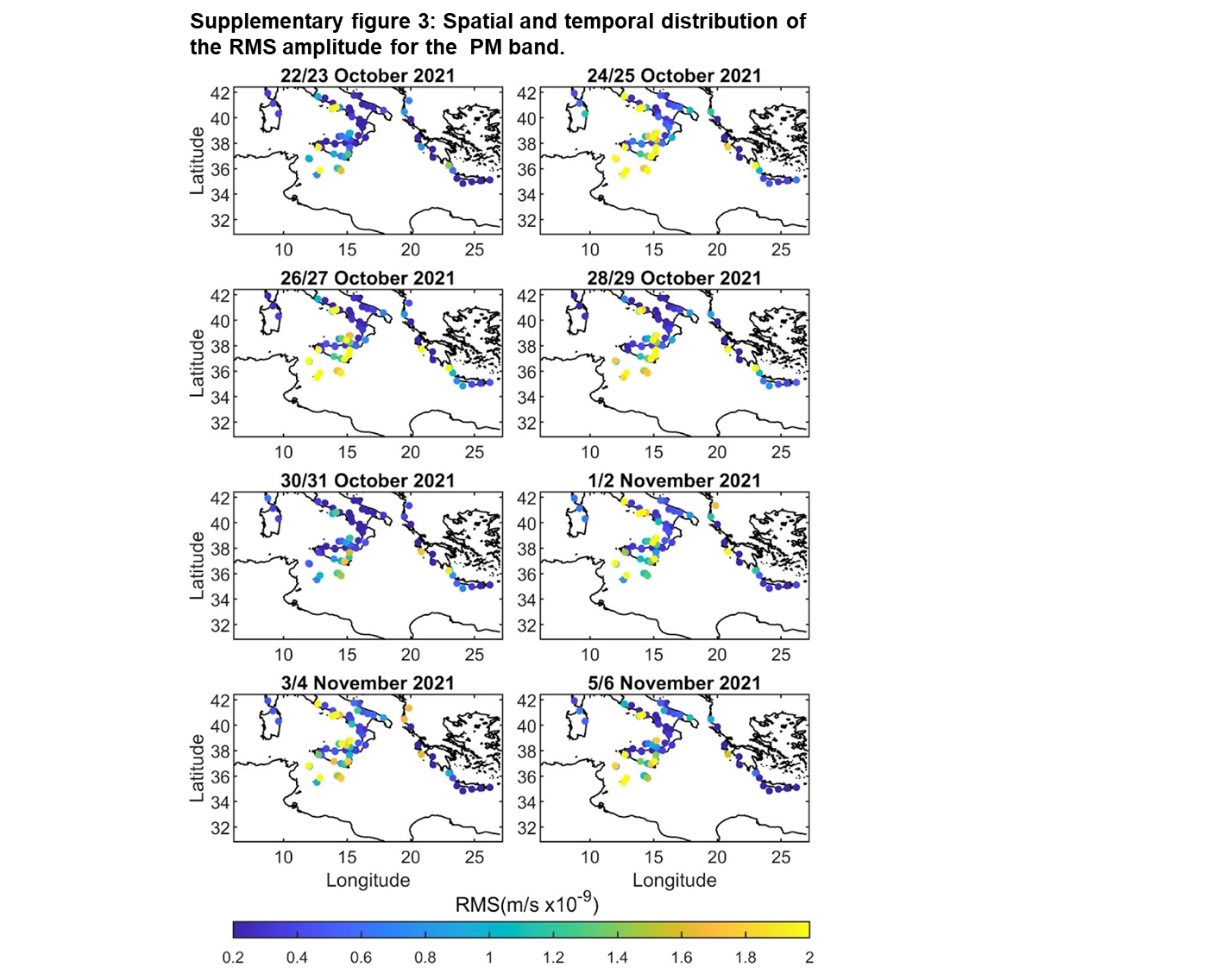


**Supplementary figure 3.** Spatial and temporal distribution of the RMS amplitude for the PM band. Each dot represents a station while the colors represent the RMS amplitude as specified in the color bar.


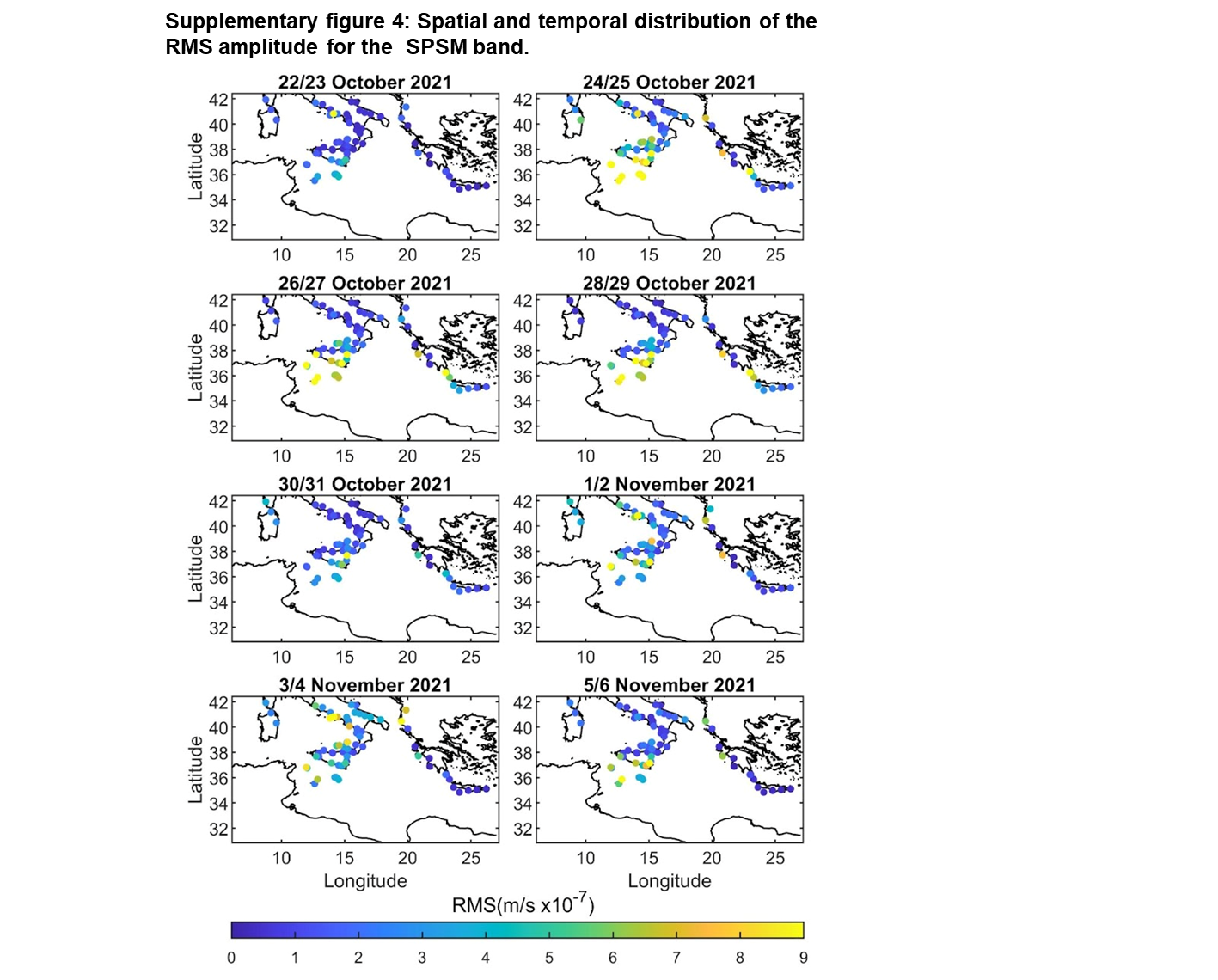


**Supplementary figure 4.** Spatial and temporal distribution of the RMS amplitude for the SPSM band. Each dot represents a station while the colors represent the RMS amplitude as specified in the color bar.


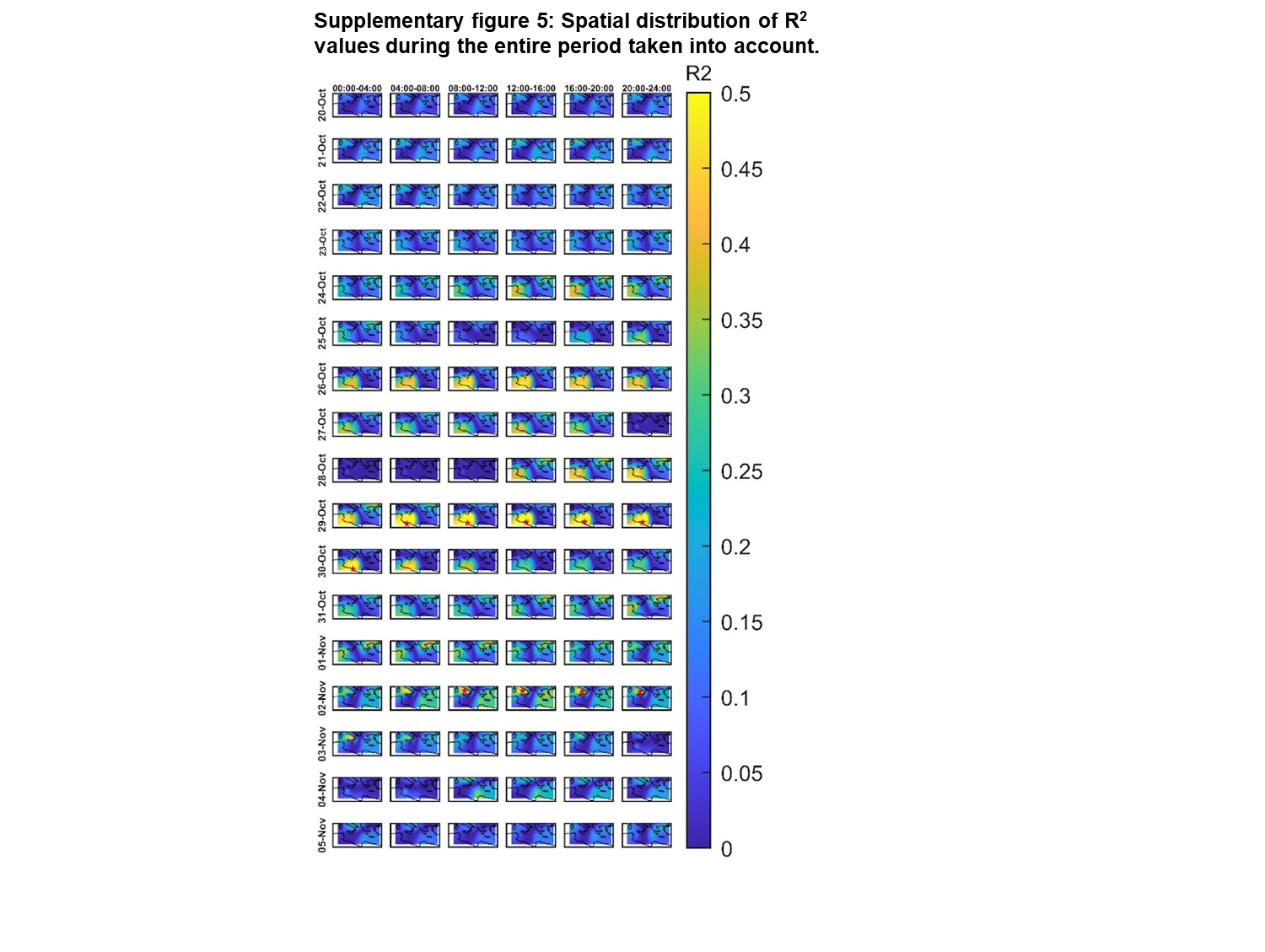


**Supplementary figure 5:** Spatial distribution of R^2^ values during the entire period taken into account. The plot rows show the changes of R^2^ values during the same day while in the columns different days are represented.


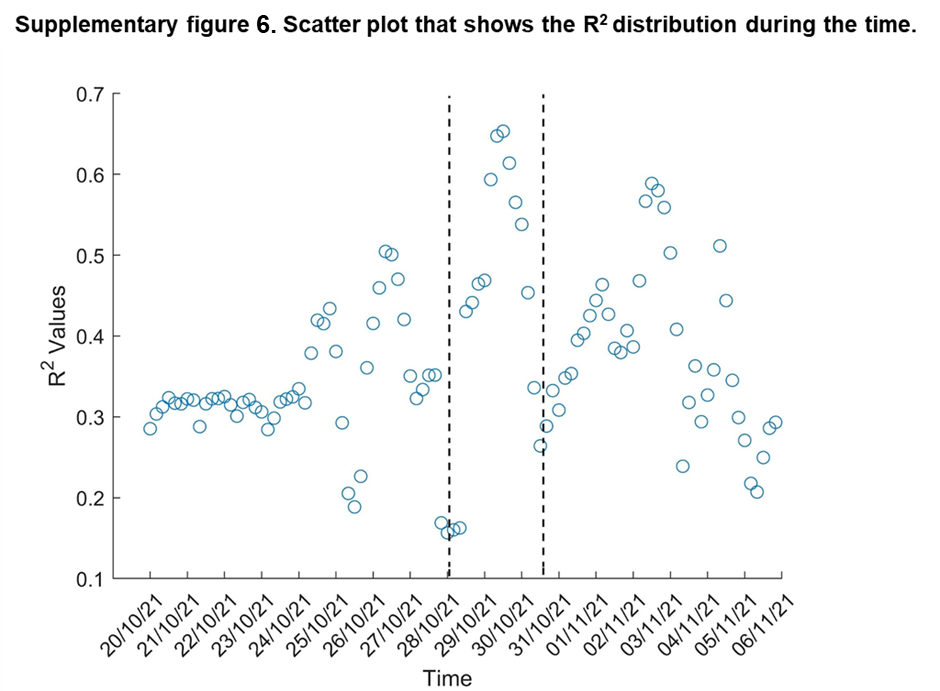


**Supplementary figure 6.** Scatter plot showing the R^2^ distribution during the time. The black dashed lines represent the time interval when Apollo reached his highest intensity.


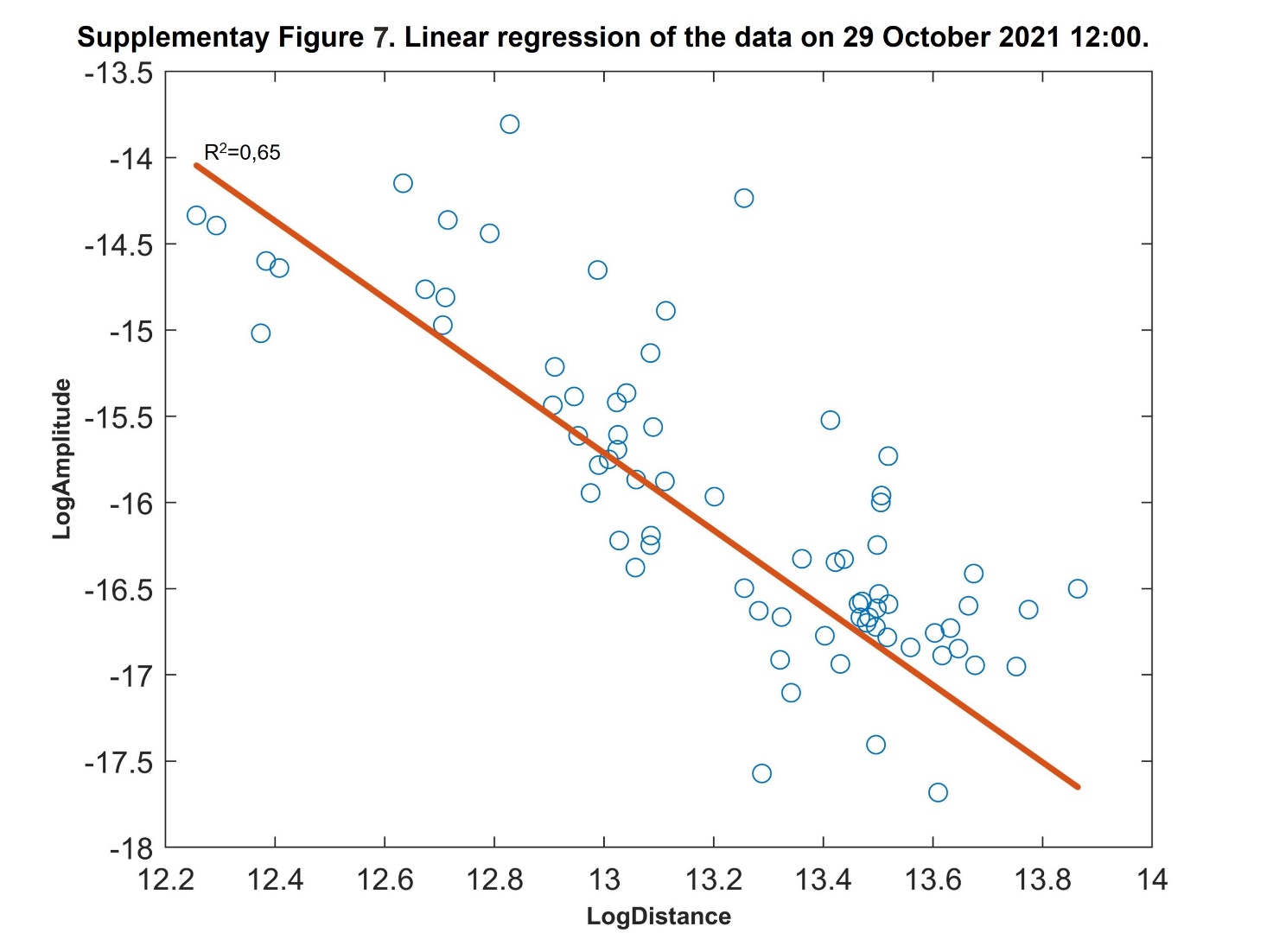


Supplementary Figure 7. Linear regression of the data on 29 October 2021 12:00 AM. The logarithm of the distance source-station and the logarithm of the amplitude are shown in the x-axis and y-axis respectively. The orange line represents the linear regression. For this regression the R^2^ value of 0.65 was obtained.


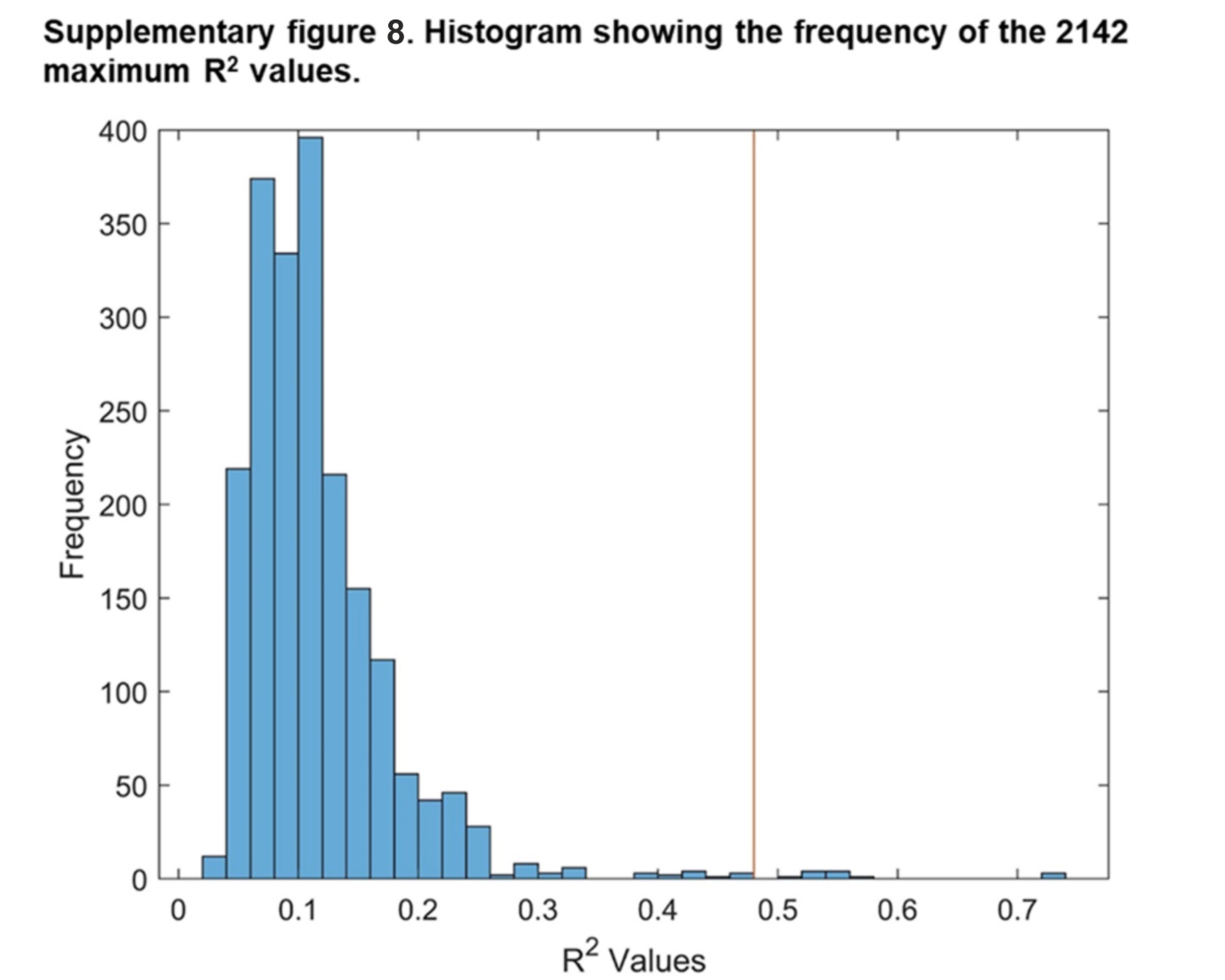


**Supplementary figure 8.** Histogram showing the frequency of the 2142 maximum R^2^ values obtained by randomly shuffling the RMS amplitude values among the stations. The red line indicates the R^2^ value corresponding with the 99th percentile.
